# High Prevalence of Genetically Related *Clostridium Difficile* Strains at a Single Hemato-Oncology Ward Over 10 Years

**DOI:** 10.3389/fmicb.2020.01618

**Published:** 2020-07-20

**Authors:** Edyta Waker, Filip Ambrozkiewicz, Maria Kulecka, Agnieszka Paziewska, Karolina Skubisz, Patrycja Cybula, Łukasz Targoński, Michał Mikula, Jan Walewski, Jerzy Ostrowski

**Affiliations:** ^1^Department of Clinical Microbiology, Maria Skłodowska-Curie National Research Institute of Oncology, Warsaw, Poland; ^2^Department of Genetics, Maria Skłodowska-Curie National Research Institute of Oncology, Warsaw, Poland; ^3^Department of Gastroenterology, Hepatology and Clinical Oncology, Centre for Postgraduate Medical Education, Warsaw, Poland; ^4^Department of Lymphoproliferative Diseases, Maria Skłodowska-Curie National Research Institute of Oncology, Warsaw, Poland

**Keywords:** infection, WGS, *Clostridium difficile*, Ion Torrent, NGS

## Abstract

**Aims:** *Clostridium difficile* (*C. difficile*) infection (CDI) is the main cause of healthcare-associated infectious diarrhea. We used whole-genome sequencing (WGS) to measure the prevalence and genetic variability of *C. difficile* at a single hemato-oncology ward over a 10 year period.

**Methods:** Between 2008 and 2018, 2077 stool samples were obtained from diarrheal patients hospitalized at the Department of Lymphoma; of these, 618 were positive for toxin A/B. 140 isolates were then subjected to WGS on Ion Torrent PGM sequencer.

**Results:** 36 and 104 isolates were recovered from 36 to 46 patients with single and multiple CDIs, respectively. Of these, 131 strains were toxigenic. Toxin gene profiles *tcdA(+);tcdB(+);cdtA/cdtB(+)* and *tcdA(+);tcdB(+);cdtA/cdtB(-)* were identified in 122 and nine strains, respectively. No isolates showed reduced susceptibility to metronidazole and vancomycin. All tested strains were resistant to ciprofloxacin, and 72.9, 42.9, and 72.9% of strains were resistant to erythromycin, clindamycin, or moxifloxacin, respectively. Multi-locus sequence typing (MLST) identified 23 distinct sequence types (STs) and two unidentified strains. Strains ST1 and ST42 represented 31 and 30.1% of all strains tested, respectively. However, while ST1 was detected across nearly all years studied, ST42 was detected only from 2009 to 2011.

**Conclusion:** The high proportion of infected patients in 2008–2011 may be explained by the predominance of more transmissible and virulent *C. difficile* strains. Although this retrospective study was not designed to define outbreaks of *C. difficile*, the finding that most isolates exhibited high levels of genetic relatedness suggests nosocomial acquisition.

## Introduction

*Clostridioides* (*Clostridium*) *difficile* (*C. difficile*) is an anaerobic, Gram-positive, spore-forming, and toxin-producing bacillus identified as the main cause of healthcare-associated infectious diarrhea (Carroll and Bartlett, 2011; Rodríguez Garzotto et al., 2015; Czepiel et al., 2019). The incidence of asymptomatic colonization by *C. difficile* may reach up to 20% during the first days of hospitalization, and even 50% after a month of hospitalization (Czepiel et al., 2019). Colonization with toxigenic strains increases the risk of symptomatic infection by nearly 6-fold; the clinical features of *C. difficile* infection (CDI) are restricted to patients with replicating bacteria that produce enterotoxin A and cytotoxin B (Denève et al., 2009). The risk of CDI in hospitalized patients is linked to case-associated areas, length of hospital stay, antibiotic treatment, and advanced age (Freifeld et al., 2011; Zacharioudakis et al., 2015; Fuereder et al., 2016; Hebbard et al., 2016; Park et al., 2016; Cornely et al., 2018; Shoaei et al., 2019).

Diarrhea in cancer patients can have non-infective and infective etiologies. Non-infective etiologies include mucosal injury caused by cytotoxic chemotherapy or radiation, however, infectious diarrhea relates mostly to CDI arising from neutropenia requiring treatment with antibiotics (Carroll and Bartlett, 2011; Alonso et al., 2012; Alasmari et al., 2014). The risk of developing CDI is 8- to 10-fold higher during a period of antimicrobial therapy and during the 4 weeks following cessation of therapy; the risk remains 3-fold higher for the next 2 months (Czepiel et al., 2019). Rates of CDI in cancer patients subjected to prolonged antibiotic treatment, chemotherapy, and/or radiotherapy; rates in those subjected to frequent or prolonged hospitalization; and rates in those with a depressed immune response (Louie et al., 2013; Park et al., 2016; Shin et al., 2016; Smits et al., 2016; Cornely et al., 2018; Shoaei et al., 2019) are approximately two times higher than those of the general hospital population (Kamboj et al., 2012). Unrecognized CDI in cancer patients may lead to serious morbidity and mortality. Patients with hematological malignancies hospitalized for chemotherapy or hematopoietic cell transplantation are exposed to multiple, concomitant risk factors that increase the risk of CDI; these patients suffer more adverse outcomes if infected (Chakrabarti et al., 2000; Chopra et al., 2011; Alonso et al., 2012; Vaughn et al., 2018). However, evaluation of CDI risk is often challenging because the incidence of CDI among hospitalized cancer patients differs nationwide (Costa et al., 2017).

Although there are no definitive criteria for definition of relatedness (Cho et al., 2020), genetic heterogeneity of *C. difficile* can be analyzed by a number of typing methods, which can be divided into band-based and sequence-based approaches (Mancini et al., 2018). The most commonly used band-based approach is the PCR ribotyping. According to the Webribo-database, which offers a standardized nomenclature for *C. difficile*, 15655 different PCR-ribotypes (RTs) were established among 55348 samples from 47 countries (Indra et al., 2008). The multilocus sequence typing (MLST) is a widely used sequence-based technique employing nucleotide sequences of housekeeping gene fragments. Unique combinations of alleles assigned to Sequence Type (ST) numbers have been grouped by their evolutionary relationships into six distinct phylogenetic clades (1-5 and C-I) (Janezic and Rupnik, 2015). In clades 1, 4, and 5, toxigenic strains were commonly combined with non-toxigenic strains, and clade C-I is associated only with non-toxigenic strains; clade 1, the largest and the most heterogenous group contained diverse STs (Knight et al., 2015; Liu et al., 2018). Some STs corresponds to a single RT, other STs corresponds to multiple RTs, and RT not always may predict the ST (Griffiths et al., 2010). While MLST and PCR ribotyping were similar in discriminatory abilities, both methods are useful for large-scale analysis, and combined strain nomenclature is often based on more than one typing, none of these can discriminate between genetically monomorphic lineages, such as those from the epidemic *C. difficile* 027 RT/ST1 clade (Kumar et al., 2016; Michael Dunne et al., 2018).

Whole genome sequencing (WGS) provides the most detailed level of bacterial genotyping, allowing the highest resolution of microbial spread. However, WGS-based typing of *C. difficile*, based on single nucleotide variants (SNVs) and on allelic profiling of core genome genes, named core genome MLST (cgMLST), is still hampered by the lack of standardized nomenclature (Bletz et al., 2018). Despite this, using WGS of *C. difficile* seems to be of practical importance in clinical settings as exemplified by several reports (Griffiths et al., 2010; Bletz et al., 2018; Kociolek et al., 2018; Mancini et al., 2018; Michael Dunne et al., 2018; Pightling et al., 2018; Aoki et al., 2019; Berger et al., 2019; Janezic and Rupnik, 2019; Kong et al., 2019; Cho et al., 2020). As recently reported, WGS better differentiates *C. difficile* relapse from reinfection than do definitions based on timing of recurrence (Cho et al., 2020).

Here, we examined the prevalence of genetically related toxigenic *C. difficile* strains to assess the long-term (over 10 years) persistence of *C. difficile* strains on a single hemato-oncology ward.

## Materials and Methods

The study was approved by the Maria Skłodowska-Curie National Research Institute of Oncology Ethics Committee (number 40/2018).

### *Clostridium difficile* Infection Screening

Between 2008 and 2018, all patients hospitalized at the Department of Lymphoma with healthcare-associated diarrhea (defined as ≥3 stools within a 24-hour period arising over the third day after hospital admission) underwent testing at the Department of Clinical Microbiology to detect pathogenic *C. difficile* toxins A and B. Tests were performed using the *C. difficile* TOX A/B kit (TechLab).

### *Clostridium difficile* Isolates

Stool samples positive in the toxin test were serially diluted and plated on selective *Clostridium difficile* agar (CLO) (bioMérieux, Marcy-l’Étoile, France) containing cycloserine, cefoxitin, and amphotericin B. Colonies were re-cultured on Columbia agar. Identification of *C. difficile* was based on colony morphology (yellowish/white, with a ground-glass appearance), a typical horse-like odor, and Gram staining. Confirmation was provided by API 20A ANA (bioMérieux) tests and MALDI-TOF MS (Bruker). Part of isolates were stored either at −80°C in tryptose-soy broth containing 10% glycerol or in Microbank tubes (Pro-Lab Diagnostic, United Kingdom).

### Antimicrobial Susceptibility Testing

Isolates were tested for susceptibility to metronidazole, moxifloxacin, vancomycin, erythromycin, clindamycin, and ciprofloxacin using ETEST strips (bioMérieux). Inoculum preparation, inoculations, and incubations followed the 15-15-15 rule, as recommended by the European Committee on Antimicrobial Susceptibility Testing. Plates were incubated at 37°C for 48 h in an anaerobic atmosphere. To detect possible metronidazole heteroresistance, plates containing metronidazole strips were incubated for five additional days under the same conditions. The selected minimum inhibitory concentration (MIC) values were as follows: ≥2 μg/ml for vancomycin, metronidazole, and clindamycin; ≥4 μg/ml for moxifloxacin; ≥8 μg/ml for erythromycin; and ≥32 μg/ml for ciprofloxacin. MIC testing was repeated twice for all strains. Reference strains *Bacteroides fragilis* NCTC 11295, *Bacteroides thetaiotaomicron* ATCC 20741, *Escherichia coli* ATCC 25922, and *Staphylococcus aureus* ATCC 25923 were always included in the tests.

### DNA Preparation, Sequencing, and Sequence Read Mapping

Subcultured single colonies from 140 available culture-positive isolates were subjected to WES. Of these, 36 isolates were recovered from patients with a single CDI, 18 women and 18 men with a median age of 55 years (ranging between 20 and 78 years), of whom 2, 30, and 4 had Hodgkin lymphoma, B-cell and T-cell non-Hodgkin lymphoma, respectively, and 104 were recovered from 46 patients with multiple CDIs, 19 women and 27 men with a median age of 47 years (ranging between 21 and 88 years) of whom 1, 36, and 9 had Hodgkin lymphoma, B-cell and T-cell non-Hodgkin lymphoma, respectively.

Genomic DNA was extracted from the isolates using a QIAamp DNA Mini Kit (Qiagen). DNA quantification was performed using a NanoDrop ND-1000 spectrophotometer. Genomic DNA libraries were prepared using the Ion Xpress Plus^TM^ Fragment Library Kit. Briefly, genomic DNA was enzymatically digested to obtain fragments of about 400 bp (Ion Shear^TM^ Plus Reagents Kit). Next, DNA fragments were purified using AgencourtTM AMPureTM XP Reagent. Adapter P1 containing barcodes was ligated, the reaction products were purified, and size selection was performed using an E-gel Size Select system and 2% Agarose gels. Products (400 bp) were cut from the gel and eluted. Next, libraries were amplified, purified, and quantified on an Agilent Bioanalyzer using a High Sensitivity DNA kit. Libraries were diluted to 100 pM. WGS was performed on the Ion Torrent Personal Genome Machine (PGM) platform using an Ion PGMTM Hi-QTM View OT2 Kit, an Ion PGM Hi-Q View Sequencing Kit, and an Ion 318TM Chip v2 BC.

Draft genomes (contigs) were assembled using MIRA5 (Sequence assembly with Mira 5, 2020), with Ion Torrent-specific settings. Strain typing was performed using schema described by Griffiths et al. (2010), and the following genes were identified using srst2 (Inouye et al., 2014): tcdA encoding toxin A (*TcdA*); *tcdB* encoding toxin B (*TcdB*); *cdtA* encoding binary toxin A (*CdtA*); *cdtB* encoding binary toxin B (*CdtB*); *tcdC* encoding the negative regulator of the *tcdA* and *tcdB* genes; *gyrA* and *gyrB* encoding DNA gyrase subunits A (GyrA) and B (GyrB), respectively (to analyze quinolone resistance-determining regions). The gene sequences were derived either from the PubMLST *Clostridium difficile* database^1^ or from the Virulence Factors database^2^. Strain typing of assembled contigs was also conducted using MLST software (Seemann, 2019). Simpson’s index of diversity (1-D) was computed to measure overall strain diversity. GoeBURST algorithm was used to determine the clusters of related STs (Francisco et al., 2009).

### Single Nucleotide Polymorphism Analysis and Phylogenetic Analysis

The reads were mapped to the *Clostridium difficile* 630 genome^3^ using TMAP (iontorrent/TS, 2020). Variant calling and distance matrix computation were then performed using the CFSAN SNP Pipeline (Davis et al., 2015), with default parameters and VarScan as the variant caller of choice. Regions with maximum of 3, 2, and 1 SNPs for 1000, 125, and 15 for each isolate were considered for this analysis. Phylogenetic trees were constructed using IQTREE (Nguyen et al., 2015), with Ultrafast bootstrap (Hoang et al., 2018) as a method of branch testing and a HKY+F+R20 nucleotide substitution model. Branches with support <95% were removed from trees using ITOL.

### Statistical Analysis

Logistic regression was used to verify whether antibiotic resistance genes and mutations increase the risk of infection’s recurrence. Spearman’s correlation coefficient was computed in order to determine association between temporal and SNP distance.

## Results

### Clinical Characteristics of the Patients

Between 2008 and 2018, 2077 stool samples were obtained from patients hospitalized at the Department of Lymphoma, Cancer Center-Institute, who were suffering from clinically significant diarrhea, abdominal pain, cramps, fever, and leukocytosis within 3 days after hospital admission. Of these, 618 were positive for pathogenic *C. difficile* toxins A and B. The number of samples sent for testing in each year ranged from 122 to 251. The number of confirmed CDIs fell during the sampling period, with median positivity rates of 48.8, 30.3, and 10.5% in 2008–2010, 2011–2014, and 2015–2018, respectively (Table 1). The majority of CDI episodes were considered to be hospital-onset healthcare-acquired; malignancy was a comorbidity in all patients with CDI.

**TABLE 1:** Number of samples tested and the percentage of confirmed *Clostridium difficile* infections (CDIs).

Year	Samples tested	CDI confirmed
2008	122	59 (48.8%)
2009	200	116 (58%)
2010	203	93 (45.8%)
2011	223	77 (34.5%)
2012	147	37 (25.2%)
2013	251	96 (37.8%)
2014	229	61 (26.6%)
2015	208	31 (15%)
2016	156	12 (7.7%)
2017	134	12 (8.9%)
2018	204	24 (11.8%)

### Summary of WGS and Assembly

Because *C. difficile* culture was not part of routine microbiological diagnostics at our Cancer Center, only 285 isolates were recovered from 618 toxin-positive stool samples: of these, half of isolated bacteria were stored frozen until further use, and 140 were available for sequencing. Clinical characteristics of the patient groups whose *C. difficile* isolates were available for bacterial whole genome sequencing are presented in Table 2.

**TABLE 2:** Clinical characteristics of patients for whom *Clostridium difficile* isolates were available for sequencing.

	Patient with multiple CDIs	Patients with a single CDI
Median age in years; range	47; 21–88	56; 20–78
Male (%)	27 (59%)	19 (53%)
Diffuse large B-cell lymphoma	21 (46%)	19 (53%)
T-cell lymphoblastic lymphoma	6 (13%)	2 (5%)
Burkitt lymphoma	9 (20%)	1 (3%)
Other hematologic malignancy	10 (22%)	14 (39%)
Chemotherapy in the infection period	46 (100%)	36 (100%)

The median of the mean read coverage was 18×; the median contig number was 1291 (range, 182–7295); the median assembly length was at 4.34 Mb; and the median N50 was 9155 (Supplementary Table S1).

First, our study data have a risk of selection bias as we were not able to include a proportion of *C. difficile* 027/ST1 isolates due to logistic and technical limitations associated with the lack of clinical, epidemiological, and movement data for certain cases, as well as the fact that genomes of certain isolates did not pass quality control measures due to the insufficient levels of genome coverage.

#### MLST Results

**FIGURE 1:**
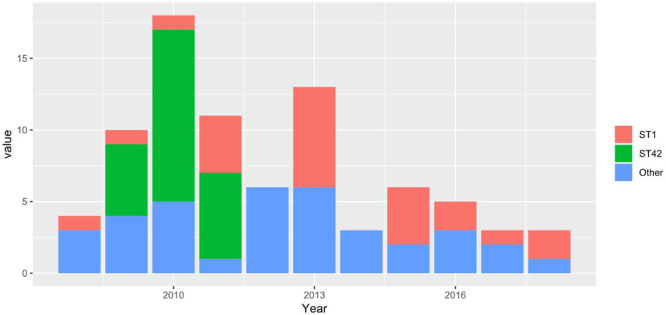
Stacked barplots, representing the number of STs in a first-time infection occurrence in a given year.

**FIGURE 2:**
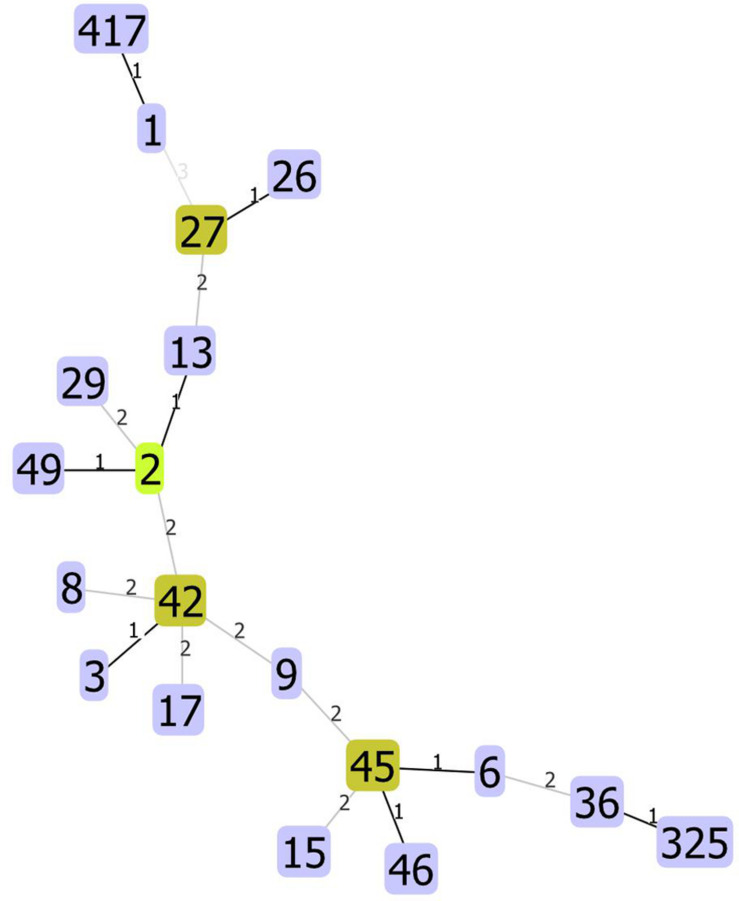
Allelic distances between STs. The STs not present on the figure have distance higher than three alleles.

We used unique combinations of alleles selected from housekeeping gene sequences, referred to as multi-locus sequence typing (MLST), which can assign *C. difficile* strains to any one of six distinct phylogenetic clades (Janezic and Rupnik, 2015). We identified 23 distinct sequence types (STs) and two unidentified strains. Two STs were dominant: ST1 and ST42 represented 31.4 and 30.1% of all strains tested, respectively. Of the 82 strains recovered from patients with a single CDI, or from patients with multiple CDIs during the first episode, 23 (28%) and 23 (28%) were ST1 and ST42, respectively. However, while ST1 strain was detected fairly consistently across all years studied, ST42 was detected only from 2009 to 2011 (Figure 1). Overall, the ecologic diversity was rather high in this dataset, as the Simpson’s index of diversity reached the value of 0.81. Strain types formed 15 clusters based on 1 allele difference distance (out of which 9 were single strain clusters) and 6 clusters based on 2 allele difference distance (out of which 4 are single strain clusters, Figure 2).

In clinical practice multiple CDIs have been classified basing on the timing of recurrence; a recurrence or a reinfection is recognized if recurrent diarrhea with confirmed infection appears within 8 weeks or beyond 8 weeks of the initial infection, respectively (McDonald et al., 2018). Of the 46 patients with multiple CDIs, 37, six and three suffered recurrent infection, reinfection, and both recurrent and reinfection, respectively, 14 suffered more than one recurrent episode. *Clostridium difficile* strains were isolated during each episode of infection from 41 patients (in total 99 isolates) and in remaining 5 patients with multiple CDIs – only at the initial infection. In 29 patients multiple infections were caused by a relapse of the original strain, and in 12 patients were caused by the newly acquired strains. Based on the infecting strain genotyping (Cho et al., 2020), the recurrent episode of infection in the latter 12 patients could be classified as a reinfection. The reinfection which occurred beyond 8 weeks of the initial infection was caused by the original strain (Supplementary Table S2). In 20 patients, the second or next CDI episode occurred during a single hospitalization, which lasted from 21 to 263 days (median = 55 days), while in the remaining patients, occurred during subsequent hospitalizations lasting from 9 to 92 days (median = 21 days).

### Toxin Gene Profiles

Although all strains studied were recovered from toxin-positive stool samples, nine subcultured single colonies from 140 culture-positive isolates appeared to be non-toxigenic strains, indicating that they might not have been responsible for CDI symptoms. WGS confirmed that all other strains were toxigenic; the predominant toxin gene profile was *tcdA+tcdB+cdtA/cdtB* (122 strains). Nine strains exhibited a *tcdA+tcdB* profile (Table 3).

**TABLE 3:** Number of sequence types (STs) and toxin gene profiles of *Clostridium difficile* isolated at the Department of Lymphoma, Warsaw, Poland, 2008–2018 (*n* = 140).

ST	Number	Toxin gene profile		
		toxA+toxB +cdtA+cdtB+	toxA+ toxB+	Non-toxigenic profile
1	44	44	0	0
11	2	2	0	0
13	4	4	0	0
15	3	0	0	3
17	1	1	0	0
2	3	3	0	0
26	2	0	0	2
27	1	0	0	1
29	2	0	0	2
3	3	2	0	1
325	2	2	0	0
35	1	0	1	0
36	5	5	0	0
37	4	0	4	0
417	3	3	0	0
42	43	43	0	0
45	2	0	2	0
46	2	2	0	0
49	1	1	0	0
5	4	4	0	0
6	4	4	0	0
8	1	1	0	0
9	1	0	1	0
NF	2	1	1	0
Total	140	122	9	9

#### Antibiotic Resistance

No isolates showed reduced susceptibility to metronidazole and vancomycin, however, all tested strains were resistant to ciprofloxacin, and 72.9, 42.9, and 72.9% of strains were resistant to erythromycin, clindamycin, and moxifloxacin, respectively. All strains belonging to ST1 and ST42 were resistant to erythromycin and moxifloxacin, whereas 56.8% of ST1 strains and 30.2% of ST42 strains were resistant to clindamycin.

Resistance to macrolides, linkosamides, and streptogramin B is caused by methylation of bacterial 23S rRNA by methylases encoded by *erm* genes (Banawas, 2018; Isidro et al., 2018). All the strains with high MIC values (>256 μg/ml) for erythromycin and clindamycin harbored *erm* genes. Of these, 19 strains were ST1, four were ST37, three were ST15, two were ST26, one was ST13, one was ST36, and one was unidentified. The *ermG* gene was present only in three isolates, ST3, ST27, and ST45. Two strains (ST3 and ST27) isolated from patients with a single CDI had high resistance to erythromycin and clindamycin. The ST3 strain, in addition to *ermG*, also harbored *msrD*, which is associated with efflux resistance to macrolides (Isidro et al., 2018).

Usually, *C. difficile* resistance to fluoroquinolones results from alternations in target structures (gyrA and/or gyrB) via nucleotide substitutions (Dridi et al., 2002; Wang et al., 2018). Here, we found that the most common mutation in gyrA was Thr82→Ile, which was detected in 68.1% of strains, all with an MIC > 256 μg/ml for ciprofloxacin and moxifloxacin. This mutation was present in all tested ST1, ST37, ST42, and ST417 strains, and in some fluoroquinolone-resistant ST29 and ST36 strains. The GyrB Asp426→Asn mutation was found in ST2, ST11, and ST15 fluoroquinolone-resistant strains, whereas gyrB Ser366→Ala was identified in the ST17 strain, and gyrB Arg447→Lys was identified in the ST417 strain. The presence of the gyrA mutation during the first infection increased the odds of subsequent infection, whereas the presence of MLS resistance genes reduced the odds of subsequent infection (Table 4 and Supplementary Table S3).

**TABLE 4:** Odds of recurrent infection by *Clostridium difficile* showing differing resistance to antibiotics.

	OR	*p*-value
AGly	0.590476	0.458958
MLS	0.330827	0.032684
Tet	0.5	0.461585
gyrA	3.541667	0.007709
gyrB	0.177778	0.130317

*OR, odds ratio; AGly, aminoglycosides; MLS, Macrolides, lincosamide, and streptogramin; Tet, tetracyclines; gyrA – gyrase A mutation, conferring resistance to fluoroquinolones, gyrB – gyrase B mutation, conferring resistance to fluoroquinolones.*

### SNP Distances and Phylogenetic Analyses

**FIGURE 3:**
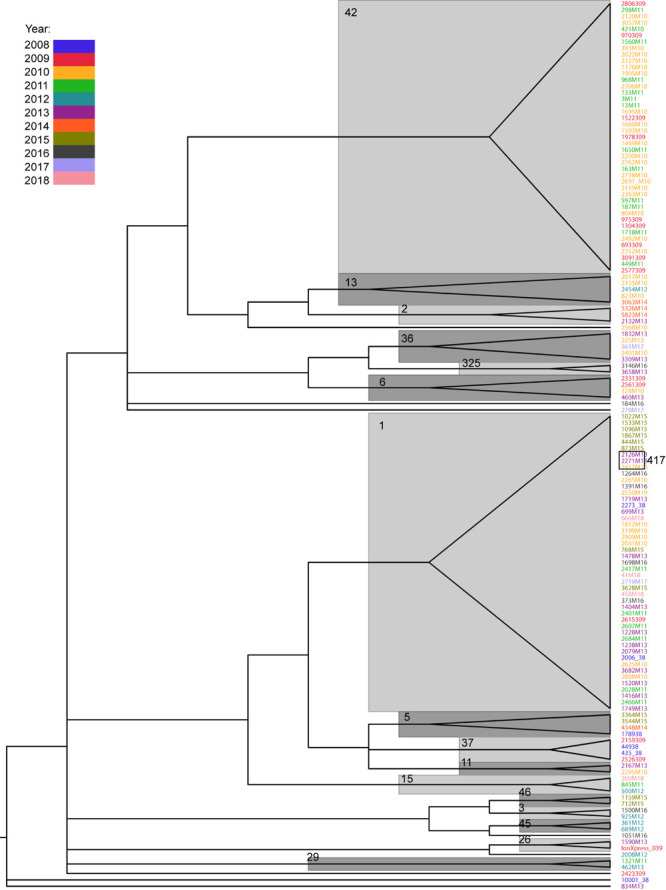
Phylogenetic tree of *Clostridium difficile* isolates, based on SNP distance matrix and computed with IQTREE. Only branches with Ultrafast bootstrap values higher than 95 are shown. Major STs are denoted near their respective branches. The years of infection are indicated in different colors.

The SNP distances within the same ST ranged from 0 to 28 for ST42 (median, 9) and 0 to 34 for ST1 (median, 12). For infections occurring in the same patient, the values were 0–22 (median, 10) for ST1 and 0–26 (median, 12) for ST42. In only four cases (one ST1 and three ST42) did the SNP distance exceed 20, which may suggest a different source of infection (Pightling et al., 2018; Supplementary Table S4). On the other hand, isolates from the same STs form on the phylogenetic tree were supported by Ultrafast bootstrap values higher than 95% (Figure 3) which suggests common source of infection.

By contrast, the SNP distances between different strains ranged from 3 to 5369 (median, 1902), confirming previous observations that the *C. difficile* genome is not well conserved (Knight et al., 2015). The SNP distance between strains did not correlate with temporal distance, neither for unrelated strains (Spearman’s coefficient = −0.08) nor for the two most common strains (Spearman’s coefficient = −0.01 and 0.06 for ST1 and ST42, respectively).

Phylogenetic tree reconstruction revealed three major nodes: the first one contains ST42 and less abundant STs (13, 2, 49, 36, 325, 6, 8, 17); the second one contains ST1 and STs 417, 5, 37, 11, and 15; and the third contains STs 46, 3, 45, and 9. The remaining strains (26, 27, 29, and 35) and one undetermined strain did not group with any other strains. Phylogenetic analysis revealed a close relationship between ST1 and ST417; this is because all ST417 bacteria share the most recent common ancestor with some ST1 strains (Figure 3).

## Discussion

Diarrhea is a prominent side effect of chemotherapy; in some cases, this can lead to dose reduction or even therapy discontinuation. The other common chemotherapy-related complication, particularly in patients with hematologic malignancies, is neutropenia accompanied by bacterial infection, which requires immediate treatment (Carroll and Bartlett, 2011). *Clostridium difficile* is the most common infectious cause of antibiotic-associated diarrhea in healthcare facilities worldwide; if unrecognized, the bacterium can cause serious morbidity and mortality in cancer patients (Alonso et al., 2012; Alasmari et al., 2014). Overall, around 14% of patients with hematological malignancies admitted to hospital for chemotherapy or hematopoietic cell transplantation are colonized with toxigenic *C. difficile* at the time of admission (Vaughn et al., 2018). Between 2008 and 2018, we collected 2077 diarrheal stool samples from patients with hematologic malignancies (mostly lymphoma and multiple myeloma) hospitalized at a single ward comprising 52 beds. Of these, 618 were *C. difficile* toxin A/B-positive. However, the percentage of toxin-positive samples fell from 50% in 2008–2010 to around 10% in 2015–2018 (Table 1).

Among 140 isolates obtained from 82 patients (36 patients with a single CDI episode and 46 with multiple CDI episodes) and subjected to sequencing, MLST identified 23 distinct STs; two strains were unidentified. The most common were ST1 (hypervirulent ribotype 027) and ST42, which accounted for 31.4% (*n* = 44) and 30.1% (*n* = 43), respectively. After eliminating multiple isolates of the same ST from the same patient, ST1 and ST42 accounted for 27% (*n* = 26) and 25% (*n* = 24), respectively, of 96 strains identified. Figure 1 shows that STs were included in three major nodes (Figure 1). According to goeBURST analysis, only 4 STs differed from the others by more than 2 alleles suggesting a close relatedness between most of isolates. However, the MLST offers rather low level of the resolution as compared to cgMLST approach (Bletz et al., 2018), which is available in MentaLiST software (Feijao et al., 2018). Unfortunately, due to the low sequencing coverage and possible sequencing errors inherent to the Torrent technology (Pereira et al., 2016) the MentaLiST could not be employed in our study.

Before 2000, RT027/ST1 was extremely rare, however, in 2000–2003 and 2003–2004, RT027/ST1 was the predominant strain identified in eight hospital CDI outbreaks in seven U.S. states, and in 12 hospitals in Montreal, Canada; the prevalence in the United States was around 51%, and that in Canada was around 84% (Loo et al., 2005; McDonald et al., 2005). By 2011, strain RT027/ST1 was still the most commonly identified strain in the United States (28.4%), and was associated with more severe disease and outcomes than other strains (See et al., 2014).

A European survey conducted in 2008 reported the prevalence of RT027/ST1 as 5% (Bauer et al., 2011), however, this has increased in several European countries in which outbreaks of severe CDI occurred (Kuijper et al., 2006). In 2013, it accounted for 30% of isolates from 32 European acute care hospitals (van Dorp et al., 2016). Similar findings were demonstrated by the EUCLID study conducted on inpatient samples from 20 European countries (samples were obtained in the winter of 2012/13 and summer of 2013) (Davies et al., 2016). Clinical CDI treatment trials conducted in 2006–09 and 2012–15 examined 1808 *C. difficile* isolates and found that the strain patterns were similar between North America and Europe (Cheknis et al., 2018). Prevalence of the RT027 strain in both the United States and Canada fell between 2011 and 2015 (Public Health Agency of Canada, 2017; CDC, 2019). Multiple outbreaks of CDI caused by the RT027/ST1 strain were driven by fluoroquinolone and rifampicin resistance (Imwattana et al., 2020; Lew et al., 2020), and a fall in its prevalence reported in England in 2010 likely resulted from restricted use of fluoroquinolones (Byun et al., 2019).

Interestingly, no RT027 strain was detected in Lebanon (Berger et al., 2018), and a multi-center study of *C. difficile* isolates in China reported only one RT027/ST1 isolate within the most predominant STs represented by clade 1 (Liu et al., 2018). It remained absent from infants, although approximately 30–40% of predominantly healthy children aged <1 year are colonized by *C. difficile* (Stoesser et al., 2017). While the *C. difficile* population shows strong genomic diversity, the core genome of ST1 strains exhibits rather low levels of genomic diversity (Steglich et al., 2015).

The Ribotyping Network (CDRN) revealed that RT106/ST42 was the second most common ribotype in England from 2007 to 2010 (Park et al., 2016); however, this strain was rarely identified (or not at all) in other European countries. However, by 2015 in North America, it had been replaced by RT027/ST1 as the most commonly identified community-associated strain, accounting for 9% of all strains (CDC, 2019). In Lebanon, RT106 was among the most prevalent RTs, accounting for 8.4% of *C. difficile* isolates (Berger et al., 2018).

Symptoms of CDIs, which range from mild diarrhea to severe pseudomembranous colitis and toxic megacolon, are caused primarily by two large protein toxins; namely, enterotoxin A and cytotoxin B, which are produced by replicating bacteria. The genes encoding these toxins, *tcdA* and *tdcB*, are located in the PaLoc region (Janezic and Rupnik, 2015). Phylogenetic analysis of whole-genome sequences representing *C. difficile* populations reveal that the PaLoc has a complex evolutionary history (Dingle et al., 2014).

Some strains of *C. difficile* produce a third toxin, termed binary toxin, which is encoded by *cdtAB*; the gene product increases adherence of *C. difficile* to epithelial cells and suppresses colonic eosinophilia (Doosti and Mokhtari-Farsani, 2014; Gerding et al., 2014; Li et al., 2018). In the early 2000s, most surveys of *C. difficile* strains reported that the prevalence of binary toxin genes was less than 10% (Gerding et al., 2014); in 2000, 5.5% of isolates at the Anaerobe Reference Unit in Cardiff were identified as positive for binary toxin genes, and the prevalence of binary toxin-positive strains in one hospital in Chicago between 1996 and 2001 was 5.8% (Geric et al., 2004). In Italy, the prevalence of binary toxin-positive strains before 1990, from 1991 to 1999, and from 2000 to 2001 was 0, 24, and 45%, respectively (Spigaglia and Mastrantonio, 2004). In 2005 and 2008, 17.2 and 23% of all toxinogenic isolates collected in 14 and 34 European countries, respectively, were binary toxin-positive (Barbut et al., 2007; Bauer et al., 2011). By contrast, binary toxin-positive strains of *C. difficile* were detected rarely in South Korea (Byun et al., 2019) and 13 other Asia-Pacific countries (Collins et al., 2020). In Iran, 12.4% of *C. difficile* strains were binary toxin-positive (Azimirad et al., 2018). Although some reports suggest that binary toxin is found preferentially in epidemic clones (Gerding et al., 2014), including ST1/ribotype 027 and ST11/ribotype 078 [which were associated with severe clinical symptoms in North America and Europe], it is possible that an increased proportion of binary toxin-positive strains may be independent from epidemic clones (Li et al., 2018).

Here, with the exception of four non-toxigenic strains (STs 15, 26, 27, and 29) and a single isolate from ST3, all isolates were toxigenic. We found that 122 (87%) of 140 isolates harbored the main toxin type, which comprised toxins A/B and a binary toxin, and only nine isolates (belonging to STs 9, 35, 37, and 45, and an unnamed ST) did not harbor the *cdtAB* gene. Notably, of three ST3 isolates, two were (*tcdA+tcdB+cdtA/cdtB*) and one was non-toxigenic. It is unclear whether transfer of the PaLoc locus is possible between toxigenic and non-toxigenic strains, however, this may have occurred here (Brouwer et al., 2013). Thus, the majority of infections were caused by toxigenic isolates carrying the *cdtAB* gene; 17 out of 82 patients died during the infection-related period. However, only in one case was death (caused by toxic megacolon) related directly to infection. In other cases, the relationship between CDI and death was not so obvious. As concluded by Gerding et al. (2014), while the binary toxin CDT may be an important virulence factor of *C. difficile* (it is associated with increased mortality, or is a marker for more virulent *C. difficile* strains), further studies are needed to determine its significance in clinical practice.

RT027/ST1 and RT106/ST42 were two the most common strains identified in our patients. While RT106/ST42 was identified only in 2009–2011, when it was the most commonly identified strain, RT027/ST1 was documented virtually every year between 2008 and 2018. To note, the prevalence of RT027/ST1 reached 48% in hospitals in Poland with an outbreak of CDI during September 2011 to August 2013 (Pituch et al., 2018). The hypervirulent RT027/ST1 contains several virulent factors, such as A/B toxins, TcdC gene mutation increasing the production of these toxins and hypersporulation increasing reproduction and spread of bacteria (Fatima and Aziz, 2019). However, a retrospective analysis by Bauer et al. exhibited that this strain was associated with a decreased odds of severe disease and did not increase in-hospital mortality or recurrence rate (Bauer et al., 2017). As reviewed recently (Fatima and Aziz, 2019) other reports also did not show its worse outcomes compared to the other strains. Thus, the widespread association of CDI with hypervirulent strains may rather result from the increased sporulation (Merrigan et al., 2010) possibly in synergy with an antimicrobial resistance, a key factor in CDI outbreaks (Imwattana et al., 2020; Lew et al., 2020). Since the quantification of sporulation was not performed in this study, we were not able to associate the endospores formation with the hypervirulence among the selected strains.

Asymptomatic carriers and symptomatic patients can excrete spores that are metabolically dormant and highly resistant to a variety of disinfectants and antibiotics; this allows them to spread by direct (person-to-person) or indirect (environmental) modes of transmission, and increases rates of recurrent CDI (Li et al., 2018). Within 2 months after treatment of an initial CDI episode, up to 33 and 45% of patients develop relapse after the first or second episode of CDI, respectively (Viswanathan et al., 2010). Also, the 027/ST1 strain secretes higher levels of toxins, and shows increased sporulation and biofilm formation (Vedantam et al., 2012). However, a severe CDI can be caused by the binary toxin-positive, non-RT027, and non-RT078 *C. difficile* strains (Li et al., 2018).

Recurrent infection may result either from reinfection caused by a newly acquired strain or from relapse caused by the original strain; 16–50% of CDI recurrences are due to reinfection with a different strain (Eyre et al., 2012). Here, of the 46 patients with multiple CDIs, 14 had more than one infection episode. Of 41 patients who were sampled during each CDI episode, isolates from 12 patients obtained during the initial and following episodes were genetically unrelated, indicating that the second or further infection was caused by newly acquired strains. In 29 patients, the isolates were clonal, indicating relapse caused by the original strain. In all patients in whom a second CDI occurred within the first 2 months of infection, the second episode was caused by the original strain (Supplementary Table S2). Thus, in agreement with other studies (Eyre et al., 2014; Mac Aogáin et al., 2015; Kumar et al., 2016; Sim et al., 2017), relapses were more common than reinfections. In fact, discriminating between relapses and reinfection had no clinical implications.

*Clostridium difficile* infection is a challenge in healthcare settings due to the expanding at-risk population and increased virulence of *C. difficile* strains that are more resistant to treatment (Shoaei et al., 2019). The high numbers of toxin-positive tests (up to 50% of diarrheal samples) in patients with hemato-oncological malignancies in 2008–2010 suggests an endemic *C. difficile* setting. In July 2010, vaporized hydrogen peroxide was used to decontaminate areas of the Department of Lymphoma, resulting in a significant decrease in the incidence of toxin-positive samples. Already in 2011, the number of toxin-positive samples fell to 34% and then to 25% in 2012. Further reductions in the percentage of positive stool samples (8–12%) occurred between 2016 and 2018. Thus, although environmental contamination of healthcare facilities may increase over time, we documented a significant level of reduced susceptibility to CDIs in recent years, which may be associated with the introduction of an epidemiological regime (including optimization of hand, furnishing, toilet and medical devices hygiene) and growing awareness of CDIs among hospital staff. In endemic settings in which standard infection prevention and controls are optimized, the burden of CDIs cannot be explained by patient–patient transmission alone (Hebbard et al., 2016; Czepiel et al., 2019).

Some studies have used genomic approaches to examine the local nosocomial epidemiology of CDIs (Griffiths et al., 2010; He et al., 2013; Bletz et al., 2018; Kociolek et al., 2018; Mancini et al., 2018; Michael Dunne et al., 2018; Pightling et al., 2018; Aoki et al., 2019; Berger et al., 2019; Janezic and Rupnik, 2019; Kong et al., 2019). A 3.6 year study involving WGS of isolates from more than 1200 patients with CDI reported that only 35% of *C. difficile* cases were genetically related (Eyre et al., 2013); another study of adult patients performed in the United Kingdom reported that 19% were genetically related (Mawer et al., 2017). A slightly higher rate of genetic relatedness was documented in the midst of a 027/ST1 outbreak in a Canadian hospital, but putative transmission among asymptomatic patients was infrequent (3%) (Kong et al., 2019). A single-center cohort of children with CDI was subjected to WGS, which identified a highly diverse group of *C. difficile* isolates. Among 131 CDIs in 107 children, 104 isolates were genetically distinct, and only eight were identified in more than one patient and two in more than two patients. Thus, direct or indirect transmission of *C. difficile* among symptomatic children is even less common than among adult patients (Kociolek et al., 2018).

The retrospective study described herein was not designed to define outbreaks of *C. difficile*, especially short-term endemic CDIs caused by ST42 strains. While most isolates exhibited high levels of genetic relatedness (within the same ST), indicating nosocomial acquisition, lack of environmental samples from inpatient and outpatient sites did not allow us to examine the presence of putatively transmitted *C. difficile* isolates. According to WGS-based molecular studies, most new cases of CDI in endemic settings cannot be explained by transmission from symptomatic cases (Freifeld et al., 2011), raising interest in the role of colonized patients in transmission of *C. difficile* in healthcare facilities and/or pre-existing colonization that transiently reaches detectable levels during hospitalization (Kumar et al., 2016). This may also be true for *C. difficile* isolates that are genetically unrelated to any others. As suggested recently, interventions to reduce the susceptibility of exposed patients to disease (including antibiotics), rather than simply reducing transmission of *C. difficile* from symptomatic patients, might have played a major role in reducing the incidence of *C. difficile* infection (Knight et al., 2015). In fact, all our patients received antibiotics, and hospitalization periods (often repeated at short intervals) ranged from 9 to 263 days. Of note, as shown in this study, the presence of the *gyrA* mutation in isolates from the first infection may predict recurrence of CDI, while the presence of MLS resistance genes may predict non-recurrence.

Our retrospective study has several limitations. Firstly, the decision to test for *C. difficile* was made as a routine practice by the physicians uninvolved in the study, and clinical data were collected from, sometimes incomplete, routine medical records with gaps in the epidemiological and clinical data. These might create a patient selection bias. Secondly, due to logistic limitations, only 140 isolates were available for sequencing which combined with the insufficient sequencing genome coverage in certain isolates could create a bias regarding a bacterial strain relatedness. Thirdly, this is a single-center cohort study which limits the generalization of the findings among other wards of our hospital.

To sum up, the high proportion of patients infected in 2008–2011 may be explained by the predominance of more transmissible and virulent *C. difficile* strains, including ST1 and ST42, which caused more symptomatic infections following contact with infected and asymptomatically colonized patients. Unfortunately, we have no definitive evidence for this.

## Data Availability Statement

The datasets presented in this study can be found in online repositories. The names of the repository/repositories and accession number(s) can be found at: https://www.ncbi.nlm.nih.gov/sra, PRJNA608241.

## Ethics Statement

The studies involving human participants were reviewed and approved by Bioethics Committee at Maria Skłodowska-Curie National Research Institute of Oncology. Written informed consent for participation was not required for this study in accordance with the national legislation and the institutional requirements.

## Author Contributions

JO and EW: conceptualization. AP, FA, and MM: methodology. MK and JO: formal analysis. EW, FA, AP, KS, and PC: investigation. ŁT, JW, and JO: resources. MK: data curation and visualization. EW, FA, MK, and JO: writing – original draft preparation. JO: supervision and funding acquisition. JO and AP: project administration. All authors contributed to the article and approved the submitted version.

## Conflict of Interest

The authors declare that the research was conducted in the absence of any commercial or financial relationships that could be construed as a potential conflict of interest.

## Footnotes

1 https://pubmlst.org/cdifficile/

2 http://www.mgc.ac.cn/VFs/main.htm

3 https://www.ncbi.nlm.nih.gov/nuccore/AM180355.1

## Supplementary Material

TABLE S1 Quality metrics for sequenced isolates.

TABLE S2 Time periods between isolates in patients with recurrent infections as well as ST indication.

TABLE S3 MLS resistance genes and gyrase A alleles identified during the first observed infection.

TABLE S4 SNP distance between the original and secondary strains isolated from patients with recurring infections.
